# Recurrent Deep Neural Networks for Real-Time Sleep Stage Classification From Single Channel EEG

**DOI:** 10.3389/fncom.2018.00085

**Published:** 2018-10-16

**Authors:** Erik Bresch, Ulf Großekathöfer, Gary Garcia-Molina

**Affiliations:** ^1^Data Science, Philips Research, Eindhoven, Netherlands; ^2^Philips Sleep and Respiratory Care, Pittsburgh, PA, United States; ^3^Department of Psychiatry, University of Wisconsin-Madison, Madison, WI, United States

**Keywords:** deep learning, recurrent networks, EEG, sleep staging, hypnogram

## Abstract

**Objective:** We investigate the design of deep recurrent neural networks for detecting sleep stages from single channel EEG signals recorded at home by non-expert users. We report the effect of data set size, architecture choices, regularization, and personalization on the classification performance.

**Methods:** We evaluated 58 different architectures and training configurations using three-fold cross validation.

**Results:** A network consisting of convolutional (CONV) layers and long short term memory (LSTM) layers can achieve an agreement with a human annotator of Cohen's Kappa of ~0.73 using a training data set of 19 subjects. Regularization and personalization do not lead to a performance gain.

**Conclusion:** The optimal neural network architecture achieves a performance that is very close to the previously reported human inter-expert agreement of Kappa 0.75.

**Significance:** We give the first detailed account of CONV/LSTM network design process for EEG sleep staging in single channel home based setting.

## Introduction

SLEEP can be formally defined as a state of reversible disconnection from the environment characterized by quiescence and reduced responsiveness usually associated with immobility. Although the precise function of sleep remains to be elucidated, it appears that sleep primarily benefits the brain (Cirelli and Tononi, 2008). Sleep is of the brain, by the brain, and for the brain (Hobson, 2005); not surprisingly the brain activity during sleep undergoes striking changes compared to that during wakefulness.

In humans, non-rapid eye movement sleep (NREM) and rapid eye movement sleep (REM) cyclically alternate with a periodicity of approximately 90 min. REM and NREM sleep occupy ~20 and 80% of total sleep time, respectively. NREM sleep includes lighter stages N1 and N2 and deep sleep N3 (also known as slow wave sleep). The sleep process can be characterized using the time dependent sleep stage dynamics which is represented using the hypnogram (see Figure 3).

Recent research evidence indicates that modulating sleep activity patterns, via peripheral stimulation at specific sleep stages can be beneficial in a wide range of contexts including memory acquisition, memory consolidation (Marshall et al., 2004, 2006) and relief from depression (Vogel et al., 1980; Landsness et al., 2011). To verify the validity of such interventions in practice requires conducting research in a larger population using automated means for online sleep staging with low latency to allow timely intervention.

In conventional clinical practice, a sleep stage for each 30-s long epoch is assigned by an expert sleep technician. This process referred to as *manual sleep staging* follows standardized rules (Iber et al., 2007) based on polysomnographic signals that include the electroencephalogram (EEG), electro-oculogram (EOG), electro-myogram (EMG), and the electrocardiogram (EKG).

The emergence of consumer sleep technologies that monitor sleep EEG (often just a single signal; Ko et al., 2015), has motivated the need for single channel EEG based automatic sleep staging. Real-time sleep staging is proposed in Kuwahara et al. (1988) using EEG, electro-oculogram (EOG), and electro-myogram signals (EMG). We consider here the option of achieving online automatic sleep staging on the basis of a single channel (or signal). Using a single signal permits to simplify the research setup and increases subject comfort.

In the research reported in this paper, we have leveraged state-of-the-art deep neural networks to automatically detect sleep stages (REM as well as NREM sleep stages N1-N3 denoting increasing sleep depth) and Wake based on a single EEG signal recorded with a sleep monitoring prototype. Designing the deep neural network architecture is critical to ensure sufficient accuracy and requires expert domain knowledge.

A multitude of automatic sleep staging methods have been devised to date, which are based on a variety of different feature extraction and classification schemes for both on-line and off-line use. We refer the reader to the recent article of Boostani et al. (2017) for comprehensive review. However, we have focused our investigation solely on single-channel, pure end-to-end deep learning solutions as suggested in Ko et al. (2015) and Tsinalis et al. (2016) and Biswal et al. (2017) and Supratak et al. (2017), while using causal recurrent neural networks to enable on-line sleep staging. Thus, we have modeled automatic sleep staging as a sequence-to-sequence mapping problem which maps an EEG time series to a sequence of sleep stages.

Specific questions addressed in this paper include:

1. Defining the optimal training sequence length and number of sleep sessions from different subjects to train the deep network,
2. The selection of regularization type,
3. The optimal network architecture, and
4. The role of demographically (age/gender) based personalization on performance.

## Methods

### Datasets

Our main in-house dataset consists of 147 overnight sleep recordings which were acquired from 29 healthy subjects (18 F, age: 37 ± 6.8 yrs. old) at home using a wearable sleep EEG/EOG prototype. Approximately five sleep recordings per subject with an average duration of 7.3 ± 3.9 h have been used. This study has been approved by the Western Institutional Review Board (WIRB).

The prototype recorded three signals: EEG (FPz), left EOG, and right EOG referenced to the right mastoid (M2). The signals were acquired at 1,000 Hz, high pass filtered using a single pole filter (0.3 Hz cutoff frequency), notch filtered at 60 Hz to remove power line noise, down-sampled to 100 Hz after applying an 8th-order 40 Hz Chebyshev Type 1 low pass anti-alias filter (Parks and McClellan, 1972), and saved for offline processing.

The saved EEG/EOG signals were used for manual sleep staging while only the EEG was used for automatic sleep staging.

Manual staging was performed by a certified sleep technician who assigned a sleep stage wake, REM, N1, N2, N3 or “unknown” (in case of poor signal quality or presence of artifacts) to each 30 s-long window. The percentages of annotated sleep stages across the whole dataset averaged over subjects are: 16.0% wake, 13.5% REM, 2.5% N1, 38.9% N2, 18.0% N3, and 11.1% unknown. These proportions are consistent with normal human sleep architecture (Carskadon and Dement, 2011).

As a secondary dataset for the experiments described in Section Performance on the SIESTA Database we use the public SIESTA database (Klosch et al., 2001), which includes 2 polygraphic sleep recordings of 294 subjects each, totaling 588 nights. The percentages of sleep stages across the whole dataset, when averaged across subjects, are: 15.0% wake, 19.1% REM, 10.2% N1, 43.6% N2, and 11.2% N3.

Since the SIESTA database does not include the FPz-M2 signal we approximated it by averaging the FP1 and FP2 signals after re-referencing to M2. We then filtered and down-sampled the signal to 100 Hz to match the first dataset. As sleep stage annotation we used the included Rechtschaffen-and-Kales (R&K) “consensus” score of the two primary annotators, and we combined the classes S3 and S4 into the single class N3. Sleep staging in SIESTA was done using the standard PSG which makes this database an appropriate benchmark for the method presented in this paper.

**Figure 1:**
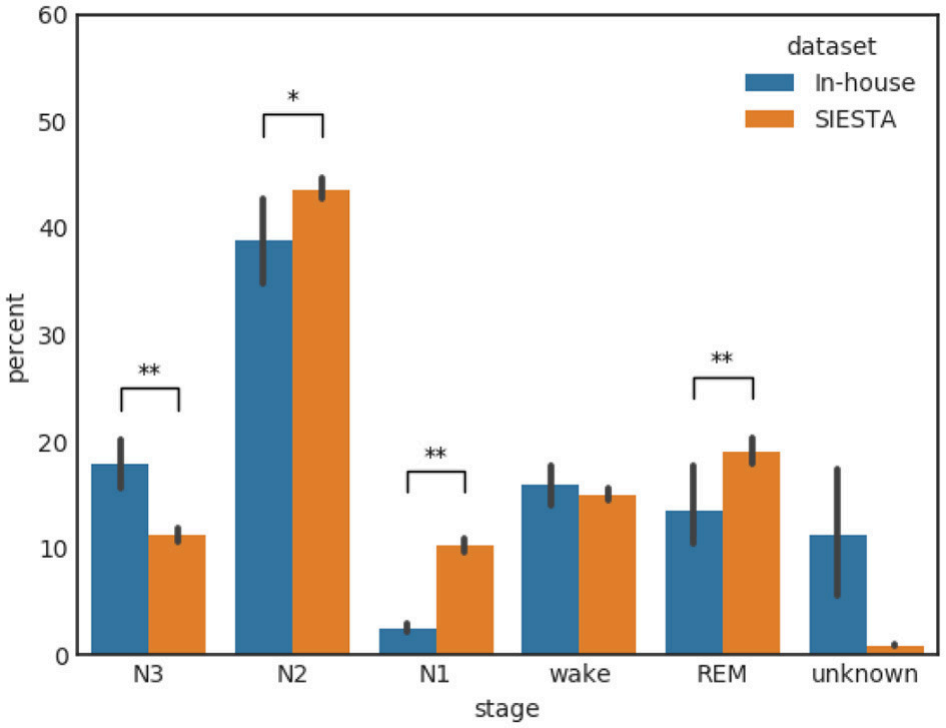
Distribution of the annotated sleep stages for the in-house database and the SIESTA database. The variance bars indicate the per-subject variance. The symbols “^*^” and “^**^” indicate a *p*-value of a Wilcoxon rank-sum test of < 0.05 and < 0.01, respectively.

Figure 1 shows the distribution of the annotated sleep stages for our primary in-house database and the secondary SIESTA database. The distributions are very similar though we find a notably larger portion of data with “unknown” label in our in-house dataset. This is consistent with the fact that our database was recorded by subjects themselves in a substantially less controlled environment compared to a sleep lab, while the SIESTA dataset was collected in professional sleep laboratories. Further, notable differences exist in the percentage of N1, N3, and REM. N1 is shorter and N3 is longer for the home based recordings compared to in-lab which may be due to the fact that subjects sleep better at home. The vertical bars in Figure 1 show the subject based variability, while the symbols “^*^” and “^**^” indicate a *p*-value of a Wilcoxon rank-sum test of < 0.05 and < 0.01, respectively.

### Baseline sleep stager network design

**Figure 2:**
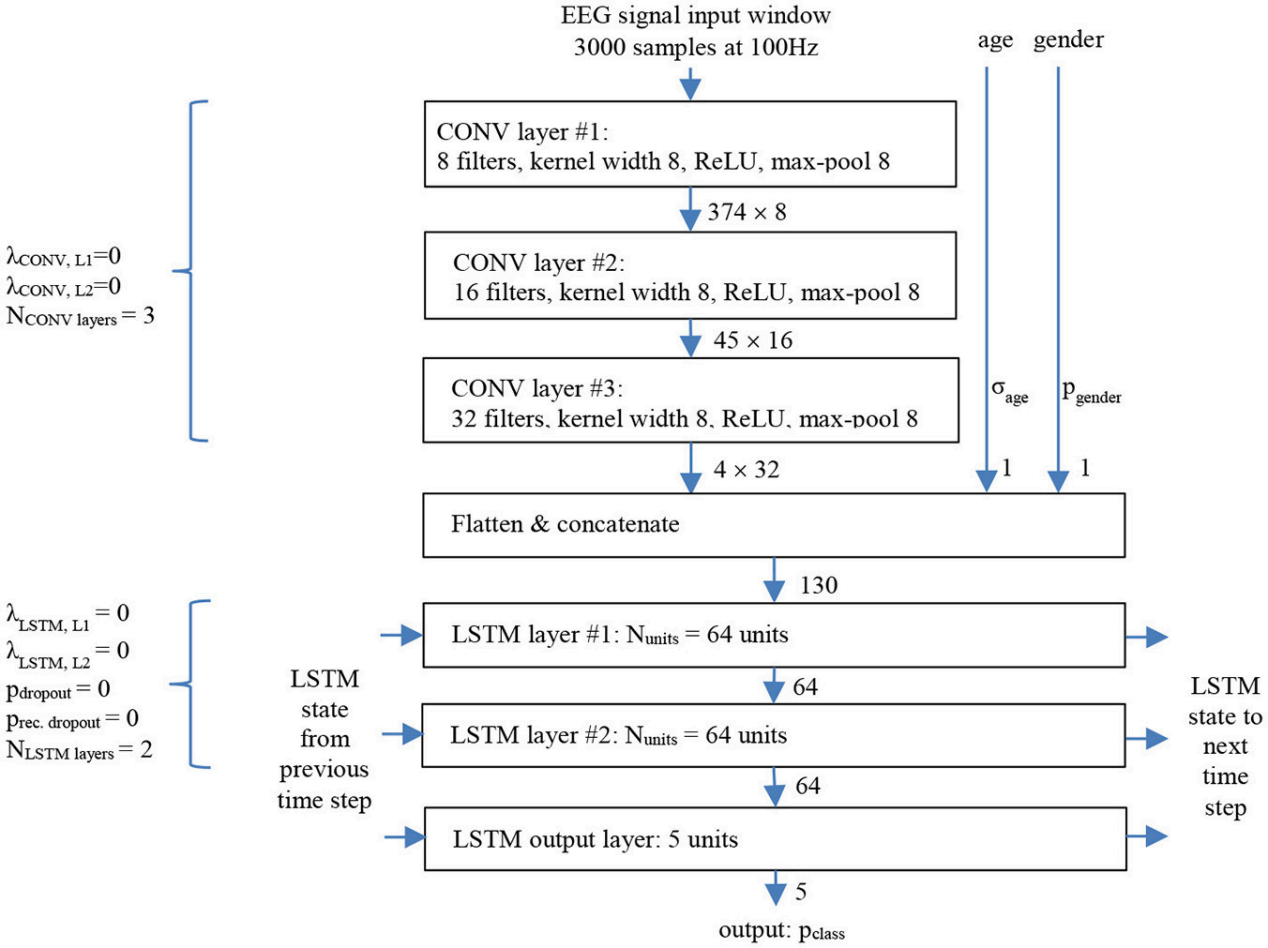
Temporally unrolled CNN/LSTM sleep stager network with baseline parameters shown for one LSTM time step, i.e., corresponding to one 30 s EEG signal window.

We consider the neural network architecture shown in Figure 2 which follows a common deep learning recipe to handle sequence models. The input signal propagates through cascaded convolutional layers (CONV; Krizhevsky et al., 2012) that act as local feature extractors, while max-pooling operations are included to allow some shift invariance. The CONV result is then processed through a series of long short term memory layers (LSTM; Hochreiter and Schmidhuber, 1997) which model longer-range temporal structure in time series data.

The network takes as input consecutive non-overlapping windows of 3,000 EEG samples (corresponding to 30 s long windows). The input is processed with a stack of 3 (N_CONVlayers_) convolutional layers, which have 8, 16, and 32 filters, respectively, kernel width 8, stride 1, no padding, rectified linear activations (ReLU; Nair and Hinton, 2010), and max-pooling by 8. The 3 convolutional layers have 72, 1,040, and 4,128 trainable parameters, and their output data dimensions are 374 × 8, 45 × 16, and 4 × 32.

The output of the convolutional layers is flattened and appended with a two-element demographic information vector: age and gender (+1 for men and −1 for women). Age and gender information are relevant as sleep architecture and EEG sleep properties vary across lifespan and that variability appears to be gender dependent (Ohayon et al., 2004; Carrier et al., 2011). In the baseline configuration both, age and gender inputs were disabled, i.e., clamped to zero.

For each EEG window “time step,” the concatenated 130-element vector is then fed to a stack of 2 (N_LSTMlayers_) large recurrent LSTM layers with 64 (N_units_) units each. The 2 layers have 50,432 and 33,024 trainable parameters, and output size 64 each.

The output layer is a small 5-unit LSTM with softmax output activation, and it has 1,400 trainable parameters. The 5-component output vector represents for each time step the *class* probabilities (or soft decisions) that the given 30 s input window belongs to sleep stages wake, REM, N1, N2, and N3, respectively. The sleep stage *hard* decision is the one associated with the maximum class probability.

It should be noted that an LSTM layer can also approximate the function of a dense feed forward layer when the memory cell functionality is disabled though the appropriate internal kernel and bias configurations. We therefore did not add a dedicated “Dense” (fully connected) layer to the output of the model.

**Figure 3:**
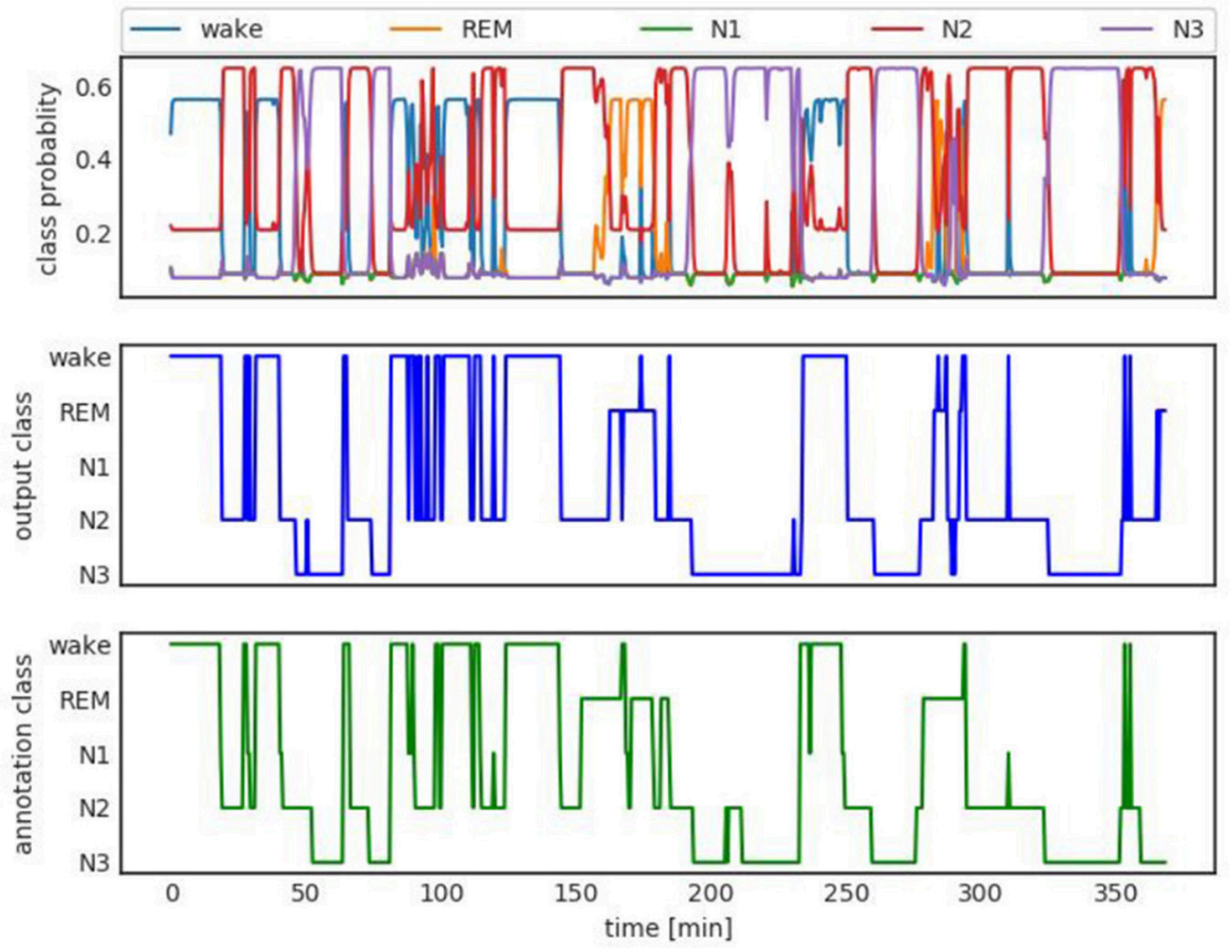
Top: Class probability output. Middle: Hard class decision. Bottom: Ground truth manual staging hypnogram. The hard decision and ground truth agree with Kappa (Cohen, 1960) 0.73.

Figure 3 shows an example of class probabilities (top panel), the corresponding hard decision (middle panel), and the manual sleep stage annotation or ground truth (bottom panel). The hard decision sleep stage output and the manual annotation agree with a Kappa score of 0.73. The Cohen Kappa statistic is a popular metric used for sleep staging (Cohen, 1960).

### Training and performance evaluation methods

The network was implemented in Python with Keras and (Chollet, 2017) and TensorFlow (Abadi et al., 2016), and it was trained with a categorical cross-entropy loss using the ADAM optimizer (Kingma and Ba, 2014; learning rate = 0.001, β_1_ = 0.9, β_2_ = 0.999, ε = 10^−8^, decay = 0). The training batch size was set to 128 sequences. The sequences are randomly picked from all training subjects. Each sequence is 128 consecutive windows long, i.e., it spans a duration of 64 min. The starting sample of a training sequence was randomly chosen from within the recording of a subject, i.e., not necessarily coinciding with the start of a 30 s annotated window, and the training target stage score was taken from the annotated stage closest to the central sample of the EEG window. This procedure is a form of data augmentation, and is described in more detail in Section Regularization. A *training epoch* consisted of 16 batches, and the training was run for 512 epochs.

We have evaluated the performance of the sleep stager using the Kappa statistic applied to the whole sleep recording and starting from the first EEG sample. Therefore, the boundaries of the windows chosen for evaluation coincide with that used in the manual scoring process.

Three-fold cross-validation (CV) is applied for all experiments reported in this paper. Folds were split across subjects, i.e., the data from a subject can either be part of the training or validation set not both. To create the folds, we partitioned our subjects into 3 groups of approximately equal size, and each group constituted the validation data for one of the 3 CV folds, while the remaining 2 groups constituted the corresponding training set.

The three-fold CV allowed us to gauge the variability of the training learning curves and the robustness against the variation of initialization conditions without creating too much of a computational burden. With a computational cost per experiment of about 6 h on a NVIDIA TESLA K80 GPU, this choice led to a total computation time of 44 days for all CV folds of all experiments. With the trained network, the computation of a single sleep stage prediction on a 30 s EEG signal epoch takes ~2 ms on a modern laptop computer with basic Python/NUMPY code.

Using the baseline network as starting point, we have investigated the effect of training data size, regularization approach, architecture choices, and subject-specific demographic information on the performance of the automatic staging network. Due to the large number of possible combinations, it is not possible to test them all. Instead we proceeded to characterize the effect of one parameter at a time while keeping the other parameters constant.

We further used the public SIESTA database for training and testing in order to study the robustness and generalization properties of our baseline sleep stager network. For comparison, we also implemented two other algorithms for sleep staging namely a logistic regression classifier using frequency domain features, and well as the convolutional neural network of Tsinalis et al. (2016).

The achieved average Kappa scores as well as their standard deviation over the three CV-folds are summarized in Table A1 in the Appendix for all experiments. The star “^*^” indicates the parameter setting used to build the baseline model, and the bold font indicates the best achieved average Kappa score over the variation of a particular experimental parameter.

## Experiments and results

### Baseline network performance

**Figure 4:**
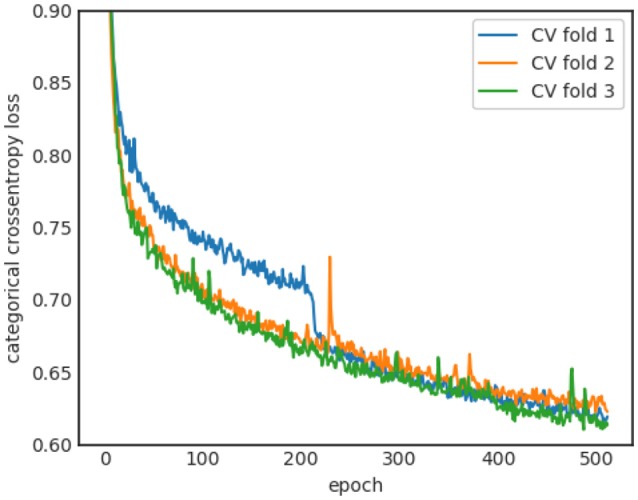
Learning curves for the three cross-validation runs.

The learning curves of the three-folds for the baseline sleep stager network are shown in Figure 4. All folds show stable convergence after 512 epochs. The average (across the three-folds) final Kappa scores are 0.885 ± 0.003 and 0.727 ± 0.005 for training and validation sets, respectively. The large gap between training and validation performance suggests overfitting effects which are tackled in the next sections.

Table 1 shows the validation confusion matrix of the baseline sleep stager, normalized and averaged across the three-CV folds. The diagonal entries are the largest except for N1 sleep which is not detected at all. This can be attributed to the fact that N1 is heavily under-represented in the dataset as it constitutes only 2.5% of the data. This however reflects the transitional nature of N1 sleep which constitutes a small portion of normal human sleep (Ohayon et al., 2004). The accuracy of N1 detection can be improved by increasing the amount of N1 data presented to the network during training as shown in Section Performance on the SIESTA Database, or by introducing class weighting in the training loss.

**Table 1:** Average confusion matrix of the baseline model on our in-house database.

**True label**	**output decision**				
	**N3**	**N2**	**N1**	**REM**	**wake**
N3	0.84	0.15	0	0	0.01
N2	0.05	0.86	0	0.05	0.04
N1	0	0.51	0	0.21	0.27
REM	0	0.11	0	0.81	0.07
wake	0.01	0.12	0	0.1	0.77

Additionally, average precision, recall and F1-scores are reported in Table 2 for each stage. N3 and N2 sleep detection have high accuracy reflected by F1-scores of 0.86 and 0.84, respectively. REM and wake stages have lower F1-scores of 0.79 and 0.76.

**Table 2:** Average per-stage performance of the baseline model on our in-house dataset.

**Stage**	**precision**	**Recall**	**F1-Score**
N3	0.87	0.84	0.86
N2	0.82	0.86	0.84
N1	0.00	0.00	0.00
REM	0.76	0.81	0.79
wake	0.76	0.77	0.76

### Insight into the type of features extracted by the convolutional layers

To qualitatively understand the nature of features extracted by the convolutional layers, synthetic single-frequency sinusoidal signals with 50 microvolt peak-to-peak amplitude (which is the average amplitude of sleep EEG) were used as input of the baseline configuration. The (4 × 32) 128 outputs (see output of CONV layer #3 in Figure 2) of the convolution layers were then evaluated for sinusoidal inputs with frequency ranging from 0.5 to 50 Hz (steps of 0.5 Hz).

**Figure 5:**
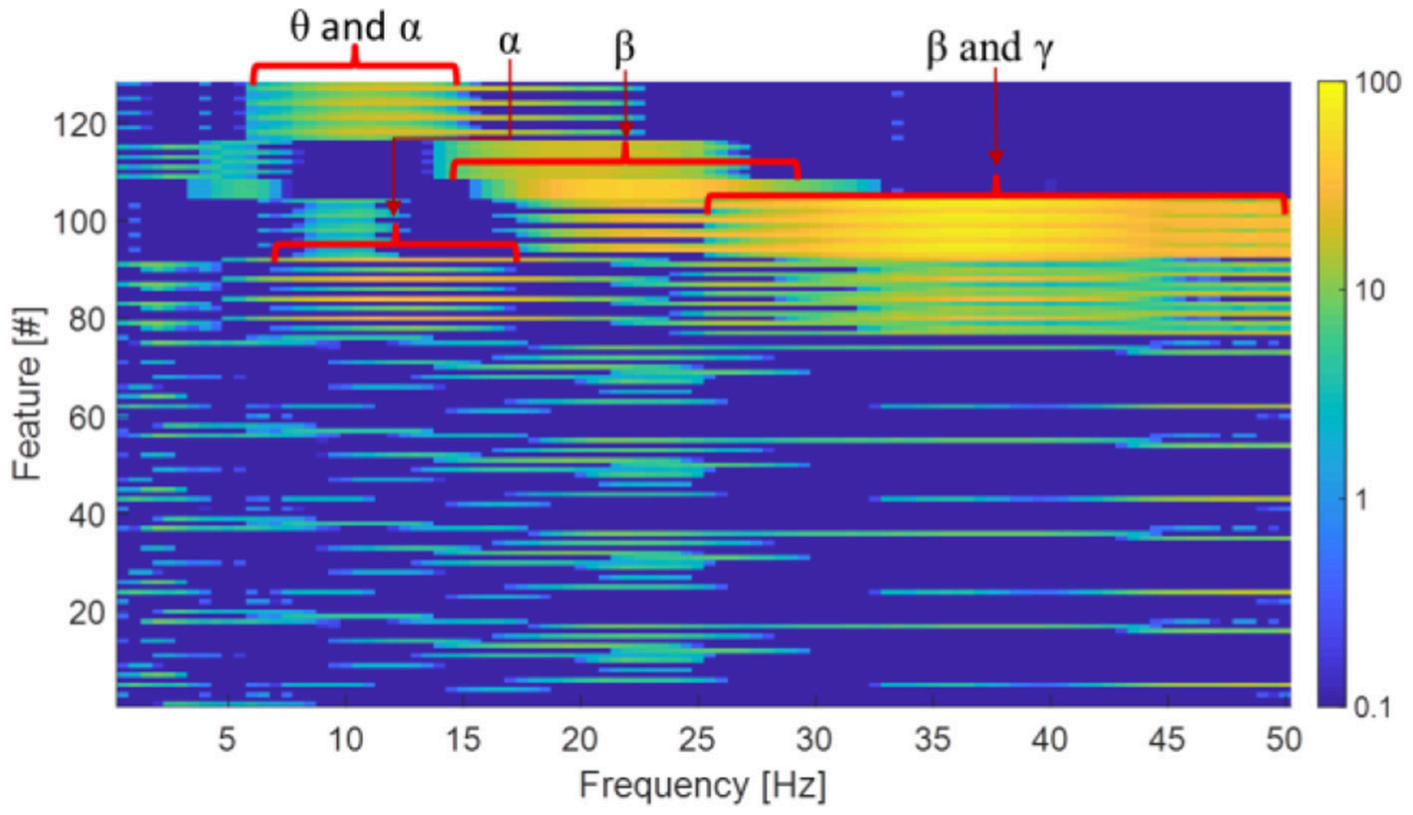
Analysis of the features extracted by the convolutional layers vs. the input frequency. For ease of visualization, feature values are shown in a log scale.

Figure 5 shows feature values for each input frequency and it can be seen that a given feature appears to respond specifically to input signals in certain frequency bands. The highlighted frequency bands (θ: 4–8 Hz; α: 8–12 Hz; β: 15–30 Hz, and γ: 30–50 Hz) shown in Figure 5 are known to be relevant for sleep.

### Influence of training data set size and sequence length

To investigate the effect of training data size, we have trained the baseline sleep stager network using a variable number (N_trainingsubjects_) of different subject datasets. N_trainingsubjects_ varied in the range from 3 to 19 subjects while maintaining all other parameters as well as the validation set the same.

**Figure 6:**
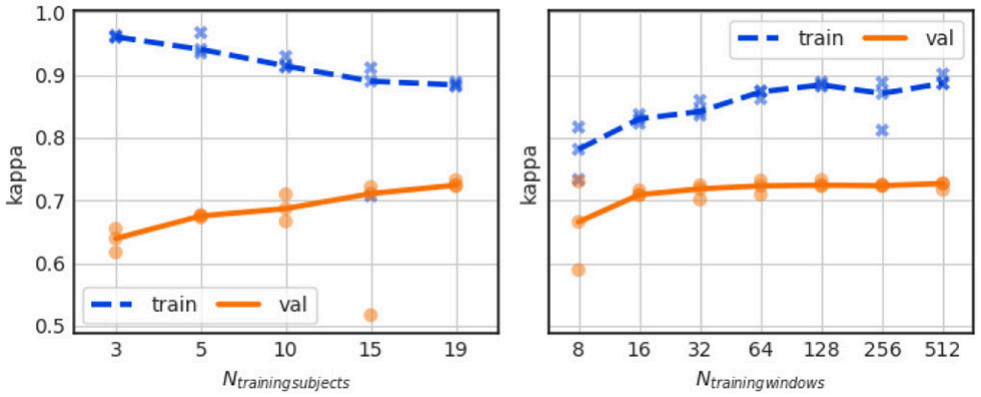
Influence of training data size (left) and training sequence length (right) on performance.

The results in Figure 6 (left) show, for each N_trainingsubjects_, the training Kappa scores (in blue “x” symbols) and the validation Kappa scores (in orange “o” symbols) for each of the 3 CV splits. The dashed blue and solid orange curves show the median Kappa (across all validation folds) for the training and validation sets, respectively. The mean and standard deviation scores for training and validation are listed in Table A1 (Exp. 1).

A reduction of N_trainingsubjects_ from 19 to 3, leads to a substantial accuracy drop from Kappa ~0.73 to 0.64. We have additionally observed that for N_trainingsubjects_ = 15, the Kappa value of one of the three CV folds converged to only ~0.5. The large difference between training and validation Kappa values suggests that our dataset (even if used in its entirety) is small for the task at hand.

As pointed out in the Introduction Section, ~90 min-long sleep cycles compose normal adult human sleep. It seems therefore reasonable to train the sleep stager network with sequences that approximately match a sleep cycle duration so that the LSTM layers can learn the long-range structure in the sleep stage data. To test the validity of this line of thought, we trained the sleep stager network with sequences of different lengths ranging from 8 windows to 512 windows, or, equivalently, 4 min to about 4 h. Hereby we left the total amount of data seen during training constant, i.e., when reducing the sequence length by a factor 2 we increased the batch size by a factor 2.

Figure 6 (right) shows the training and validation Kappa scores for training sequence lengths (N_trainingwindows_) equal to 8, 16, 32, 64, 128, 256, and 512 windows. The training Kappa score noticeably increases with longer training sequences but the validation Kappa score increases only slightly. The large gap between training and validation performance exists for all tested sequence lengths.

The average and standard deviation of the Kappa scores for this experiment are listed in Table A1 (Exp. 2). A training sequence length of 128 windows leads to the best validation Kappa score.

### Regularization

Various regularization approaches have been devised to reduce overfitting of deep neural networks, such as data augmentation, adding L1 and L2 penalty terms for the loss, and dropout (Srivastava et al., 2014). In order to address the sizable gap between training and validation performance of our baseline sleep stager model, we have studied the effect of data augmentation as well as of regularization of the convolutional layers and the LSTM layers. The results of the experiments are in Table A1 (Exp. 3 – Exp. 9).

The augmentation of multi-channel EEG data by means of spatial rotation has been proposed in Krell and Kim (2017), but this cannot be applied to single channel data. We are currently also lacking physically informed and quantitatively well understood models for other possible effects, e.g., noise sources. Therefore we resort to temporal augmentation by randomly selecting the EEG start sample of a recording during training and picking the sleep score annotations that are closest to EEG window centers. This process virtually increases the database size 3,000-fold as there are 3,000 EEG samples in an annotation window.

Clearly, the training labels obtained by this random time shift augmentation are not in correct agreement with R&K annotation rules, and they should be considered noisy. However, it has been shown in the image processing context that deep learning is very robust against label noise (Rolnick et al., 2017), and we see this confirmed also for EEG time series data as our average validation performance increases significantly from Kappa = 0.609 to 0.727. Furthermore, this method greatly reduces the variability of Kappa from ± 0.096 to ± 0.005, and hence we chose to use this augmentation as default for all experiments as was outlined in Section Training and Performance Evaluation Methods.

**Figure 7:**
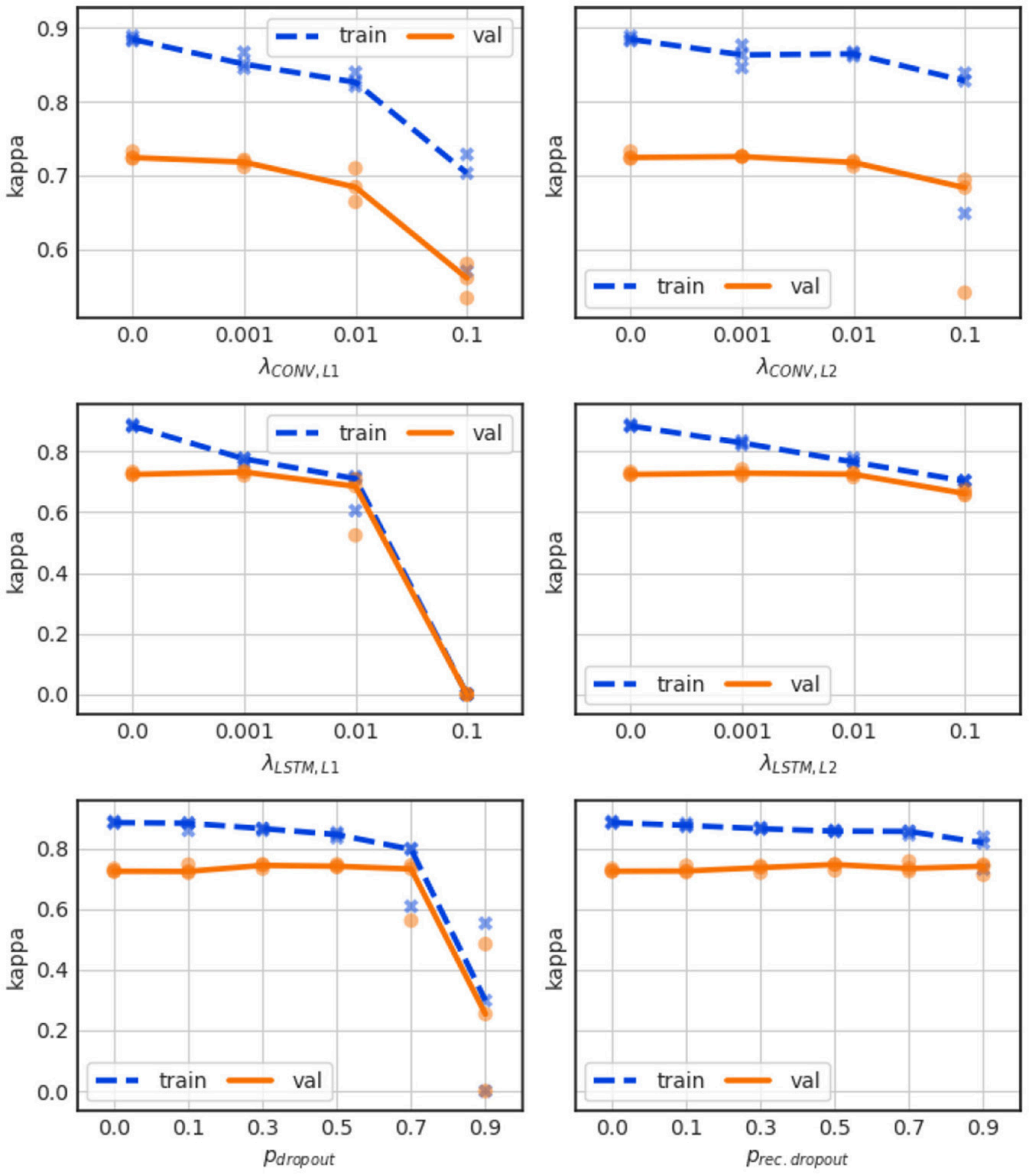
Influence of L1 penalty (top left) and L2 penalty (top right) for the convolutional layer weights, as well as for the LSTM layer weights (center left and center right). Influence of dropout for the LSTM layer forward paths (bottom left) and for recurrent paths (bottom right).

For the convolutional layers we applied separately L1 penalty and L2 penalty terms to the kernel weights. This resulted in the performance shown in Figure 7 (top left and top right, respectively). Neither mechanism closes the performance gap, and large penalties merely lead to a general loss in performance.

L1 and L2 penalties were also applied separately to the kernel weights in the LSTM layers, which constitute the majority of the network parameters, and the results are shown in Figure 7 (center left and center right, respectively). The L1 regularization closes the performance gap, but does so by largely dropping the training performance rather than increasing the validation performance.

The performance of the network is very sensitive especially to the L1 term, as a value of 0.1 totally degrades the network's training performance bringing it to a Kappa score of 0. Only a minor performance gain smaller than 1% over the non-regularized baseline model can be achieved by setting the L1 or L2 penalty weight to 0.001, which boosts the validation Kappa to about 0.73.

Dropout strategies can be applied to LSTM layers to the feed-forward paths, or to the recurrent paths (Gal and Ghahramani, 2016). The performance of the network for various forward and recurrent dropout probabilities are shown in Figure 7 (bottom left and bottom right, respectively). In both cases dropout probabilities of up to 0.5 boost the validation performance slightly, to a validation Kappa of 0.74, but still leave a large gap between training and validation performance. Very large dropout probabilities of >0.7 lead to instable training results over the 3 CV runs.

### Architecture

Many architecture choices are possible for the sleep stager network. We report our experimental results in Table A1 (Exp. 10 - Exp. 14). The experiments were conducted with shift augmentation and with three-fold CV on the entire data set.

We compared two commonly used activation functions for the convolutional layers, namely the ReLU and the leaky-ReLU (Maas et al., 2013), and two common recurrent architectures, namely the LSTM and the gated recurrent unit GRU (Cho et al., 2014). We achieve the best result with the ReLU and the LSTM.

**Figure 8:**
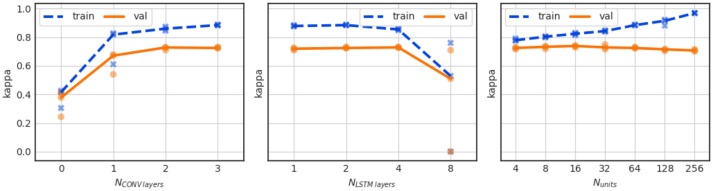
Influence of the number of convolution layers (left), the number of LSTM layers (center), and the number of LSTM units per LSTM layer (right) on performance. The LSTM configurations for the center plot are: 1 layer with 128 LSTM units, 2 layers with 64 units each, 4 layers with 32 units each, and 8 layers with 16 units each. For the right plot the LSTM stack consisted of 2 layers with N_units_ units each.

The beneficial effect on performance of the convolutional layers can be observed in Figure 8 (left), which shows the performance of the network for a varying number of layers in the convolutional stack. The performance is severely reduced if no convolutional layers are present and the EEG signal is applied to the LSTM directly. We also observe a saturation of validation performance for 3 convolutional layers, which is the number of CONV layers in the baseline network.

In the series of experiments shown in Figure 8 (center) we investigate the effect of network depth vs. width for different configurations of the LSTM stack. Here, 128 recurrent units were used for all experiments but they were configured either in a single LSTM layer, or in a 2 LSTM layer stack with 64 units each (which is the baseline configuration), in 4 layers with 32 units, or in 8 layers with 16 units each. We find that there is no appreciable benefit of network depth. On the contrary, the deepest network configuration leads to instable training results.

Furthermore, we have investigated the effect of changing the capacity of the network by varying the number of recurrent units in the 2 layer LSTM stack. As shown in Figure 8 (right), there is a large effect on the training performance (blue curve) which indicates stronger overfitting when the number of LSTM units increases from 4 to 256. The validation performance is much less affected up to 64 units per layer. A decline in validation performance appears from thereon.

We further notice a slightly increased performance over the baseline model with Kappa = 0.736 ± 0.007 for 16 LSTM units. And while the difference is statistically not significant, the 16 unit model might have been the better outset as it greatly reduces the computational complexity in the model.

### Personalization with subject specific information

The baseline sleep stager network shown in Figure 2 also accepts demographic information (age and gender of the subject), and in all prior experiments these inputs were not used, i.e., clamped to zeros.

A major concern of using personalization is overfitting because our dataset contains the data from 29 subjects only. We have therefore studied this effect in a number of experiments where we applied varying degrees of noise to the personalization information during the training. The results are summarized in Table A1 (Exp. 15 and Exp. 16).

For the age input, we added zero-mean Gaussian noise with standard deviation σ_age_, where one such noise value was chosen for every training sequence of length N_trainingwindows_ in a training batch. Similarly, with a probability p_gender_ we replaced the true gender code (+1 for men, −1 for women) by 0. Only one type of personalization was studied at a time. The other input was disabled by clamping it to zero.

**Figure 9:**
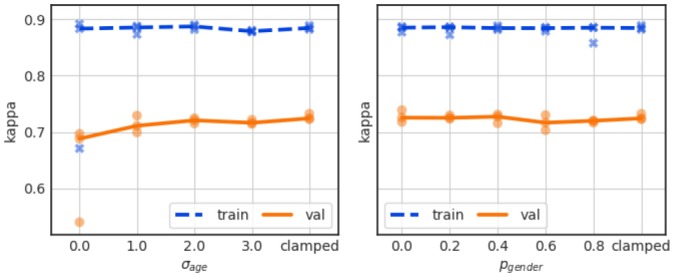
Effect of personalization with age (left) and gender (right) for varying degrees of added noise. The value “clamped” indicates that the personalization input was clamped and no information entered the network.

As shown in Figure 9 (left), age information does not lead to a performance gain when applied directly, i.e., without any regularization (σ_age_ = 0), but rather to a slight validation performance drop to Kappa just below 0.7. It is not clear if this is due to overfitting, as the training performance as largely unaffected, though we generally observe a large training/validation performance gap.

Also the use of gender information does not boost the performance, as shown in Figure 9 (right), regardless of whether the gender information is applied directly and without augmentation noise (p_gender_ = 0) or with varying degrees of noise. The Kappa curves remain flat and show a large gap between training and validation performance throughout.

### Performance on the SIESTA database

We trained our baseline network on the SIESTA database (Table A1, Exp. 17) and achieved a training Kappa of 0.797 ± 0.001 and a validation Kappa of 0.760 ± 0.022. We notice that the validation performance is slightly higher and that training-validation gap is significantly smaller than when using our in-house database. The validation confusion matrix and the per-stage performance are shown in Tables 3, 4, respectively, where the most striking difference is the improved N1 detection performance.

**Table 3:** Average confusion matrix of the baseline model on the SIESTA database.

**True label**	**Output decision**				
	**N3**	**N2**	**N1**	**REM**	**Wake**
N3	0.76	0.23	0	0	0
N2	0.03	0.91	0.03	0.02	0.01
N1	0	0.33	0.37	0.12	0.17
REM	0	0.07	0.03	0.89	0.01
wake	0	0.03	0.07	0.02	0.89

**Table 4:** Average per-stage performance of the baseline model on the SIESTA database.

**Stage**	**Precision**	**Recall**	**F1-Score**
N3	0.86	0.77	0.81
N2	0.84	0.91	0.87
N1	0.57	0.37	0.45
REM	0.84	0.89	0.87
wake	0.88	0.89	0.88

We further tested the generalization properties of our network by cross-evaluation with different databases as shown in Table 5. Here we trained and validated with the whole databases and estimated the mean and standard deviation of Kappa by boot strapping with 100 samples.

**Table 5:** Validation Kappa scores for our in-house database and the SIESTA database.

		**Validation Database**	
		**In-house**	**SIESTA**
Training database	In-house	0.727 ± 0.005	0.454 ± 0.001
	SIESTA	0.703 ± 0.002	0.760 ± 0.022

*The diagonal values were obtained with three-fold CV, the off-diagonal values from training and testing on the entire databases and boot-strapping the Kappa value with 100 samples*.

We find that training with SIESTA leads to a more transferrable (higher generalization) network (Kappa = 0.703 on our database) than training with our in-house database (Kappa = 0.454 on SIESTA).

### Comparison with other methods

We compared our approach to two other classification methods, namely a multinomial logistic regression classifier and the CNN based architecture presented in Tsinalis et al. (2016). The logistic regression based method is among the simplest approaches with the lightest computational load, while the CNN of Tsinalis et al. (2016) is the one that could be considered closest to our proposed method.

The multinomial logistic regression requires the extraction of suitable features from each 30 s epoch of the EEG signal. A wealth of linear and non-linear feature extraction methods in the time, frequency, and wavelet domains have been reported in literature for automatic sleep staging (Fell et al., 1996; Estrada et al., 2004). Frequency domain based features reflect essential properties of sleep stages therefore we have used the magnitude and phase component of the Fast Fourier Transform of the EEG signal. Since the EEG signal is real-valued this results in 3,000 independent feature values for each epoch, and they capture all information in the signal without loss. The logistic regression classifier has therefore 3,000 inputs and 5 outputs and uses 15,000 weights and 5 bias values, totaling 15,005 coefficients. The classifier achieves a validation Kappa of 0.408 ± 0.041 on our in-house dataset (see Table A1, Exp. 18).

The CNN of Tsinalis et al. (2016) consists of 2 convolutional layers, including max-pooling, 2 dense layers, and an output layer. This network requires an EEG input signal consisting of the current 30 s epoch plus the preceding 2 epochs and the 2 following epochs, thus introducing a processing delay longer than a minute. The network has 144697925 parameters in total, and it achieves a validation Kappa of 0.708 ± 0.005 on our in-house data set (see Table A1, Exp. 18).

## Discussion

The experiments reported in Section Insight Into the Type of Features Extracted by the Convolutional Layers on the training data requirements give valuable practical guidelines for an end-to-end deep learning implementation of an automatic sleep staging network based on a single EEG signal. Specifically, about 20 training subjects are a viable minimum for such a purely data driven design leading to a performance of Kappa = 0.73, which is near the reported human inter-rater agreement of about 0.75 for full-montage multi-channel EEG data (Danker-Hopfe et al., 2009). Hence the neural network sleep stager is almost indistinguishable from a human annotator even though it operates only on a single-channel EEG data.

Further, we establish that training sequence lengths much shorter than a typical sleep cycle are feasible for training state-of-the-art recurrent neural network. This eases the memory requirement, which is of great practical impact especially when the neural network is trained on a GPU with tight memory constraints.

In almost all experiments we have observed a substantial gap between training and validation performance, which failed to be closed by any of attempted regularization techniques described in Section Regularization. A possible explanation is that the intra-annotator agreement (of our single annotator) sets the upper bound on the performance. Multiple scorings of the data by the same annotator are planned as future work to validate this hypothesis.

Our experiments in Section Architecture indicate that a combination of convolutional layers and LSTM layers constitutes an appropriate network architecture for causal sleep staging of EEG data, and that high performance can be achieved with only a single EEG channel. Interestingly, we also found that relatively shallow networks with few convolutional layers and LSTM layers perform well enough, and that relatively few recurrent units are sufficient.

We further found in Section Personalization With Subject Specific Information that user specific personalization with age and gender information does not improve performance. The reason could be that the benefit of personalization information is masked by intra-annotator noise, or that the neural network automatically learns features that are robust to age and gender-related characteristics of the EEG signal.

The results of the experiments with the SIESTA database shown in Section Performance on the SIESTA Database indicate that, given such a large training database, a deep neural network sleep stager can be trained that is almost indistinguishable in performance from a human annotator.

We attribute the slight drop in performance with our in-house database to its much smaller size, the fact that it was recorded in less controlled home conditions vs. in lab environment which increases signal noise, and the possibility of having noisier ground truth annotations for validation, as only a reduced EEG montage is used. We further find that a network trained with the SIESTA database transfers well to the single-channel home setting, also if the EEG montage is different and the EEG channel has to be approximated.

The experiments in Section Comparison With Other Methods show that our CNN/LSTM approach compares favorably with the logistic regression method and the neural network of Tsinalis et al. (2016). Our validation performance on our in-house dataset is, as expected, significantly better than that of the basic logistic regression classifier with simple frequency domain features, though even the logistic regression performs better than chance. Our performance gain over the method of Tsinalis et al. (2016) is much smaller. It should be noted however, the network of (Tsinalis et al., 2016) has a significantly larger memory footprint as it requires 144 million parameters and it introduces a 3-epoch processing delay, whereas our proposed baseline CNN/LSTM network has only approximately 90,000 parameters and introduces a 1-epoch delay while also performing slightly better. Such a reduced memory requirement and low-latency characteristic can be advantageous in an embedded real-time implementation.

Various optimization strategies remain to be addressed in future research such as: (1) an alternative loss function that can express the intrinsic differences between sleep stages. Such a loss function could, e.g., penalize the confusion between N3 and N2 less than between N3 and wake. (2) Defining and combining partial loss functions for each layer of the network as proposed in Zhu and Bain (2017). (3) Sleep cycle dependent sleep stage decisions to take into account the time dependent probability of sleep stages that change between sleep cycles.

## Conclusion

A causal recurrent deep neural network consisting of CONV and LSTM layers can achieve a sleep staging performance that is very close to human inter-rater agreement. The performance is robust against age/gender based sleep variability. This result shows feasibility of high sleep staging accuracy using a single EEG signal recorded at home.

## Author contributions

EB contributed to experimental design, data analysis, and writing. UG contributed to experimental design, data analysis, and writing. GG contributed to experimental design, data collection, data analysis, and writing.

### Conflict of interest statement

The authors declare that the research was conducted in the absence of any commercial or financial relationships that could be construed as a potential conflict of interest.

## Appendix

**Table A1:** Experimental results.

**Exp**.	**Parameter**	**Value**	**Kappa_Train_**	**Kappa_*Validation*_**
1	N_*trainingsubjects*_	3	0.962 ± 0.002	0.637 ± 0.019
		5	0.948 ± 0.017	0.675 ± 0.002
		10	0.919 ± 0.009	0.688 ± 0.022
		15	0.836 ± 0.112	0.650 ± 0.115
		19^*^	0.885 ± 0.003	**0.727** ± 0.005
2	N_*trainingwindows*_	8	0.777 ± 0.042	0.662 ± 0.071
		16	0.830 ± 0.007	0.711 ± 0.004
		32	0.846 ± 0.012	0.715 ± 0.012
		64	0.870 ± 0.007	0.721 ± 0.011
		128^*^	0.885 ± 0.003	**0.727** ± 0.005
		256	0.857 ± 0.040	0.724 ± 0.001
		512	0.892 ± 0.009	0.724 ± 0.006
3	Shift augmentation	off	0.863 ± 0.142	0.609 ± 0.096
		on^*^	0.885 ± 0.003	**0.727** ± 0.005
4	λ_*CONV, L*1_	0^*^	0.885 ± 0.003	**0.727** ± 0.005
		0.001	0.854 ± 0.011	0.717 ± 0.005
		0.01	0.829 ± 0.010	0.686 ± 0.023
		0.1	0.667 ± 0.085	0.559 ± 0.023
5	λ_*CONV, L*2_	0^*^	0.885 ± 0.003	**0.727** ± 0.005
		0.001	0.862 ± 0.015	0.726 ± 0.000
		0.01	0.864 ± 0.003	0.717 ± 0.004
		0.1	0.772 ± 0.107	0.640 ± 0.085
6	λ_*LSTM, L*1_	0^*^	0.885 ± 0.003	0.727 ± 0.005
		0.001	0.773 ± 0.008	**0.729** ± 0.008
		0.01	0.678 ± 0.063	0.639 ± 0.101
		0.1	0.000 ± 0.000	0.000 ± 0.000
7	λ_*LSTM, L*2_	0^*^	0.885 ± 0.003	0.727 ± 0.005
		0.001	0.829 ± 0.006	**0.730** ± 0.011
		0.01	0.770 ± 0.008	0.723 ± 0.007
		0.1	0.703 ± 0.001	0.664 ± 0.010
8	p_*dropout*_	0^*^	0.885 ± 0.003	0.727 ± 0.005
		0.1	0.875 ± 0.014	0.730 ± 0.015
		0.3	0.863 ± 0.004	0.741 ± 0.008
		0.5	0.844 ± 0.009	**0.742** ± 0.005
		0.7	0.735 ± 0.109	0.679 ± 0.102
		0.9	0.284 ± 0.277	0.246 ± 0.242
9	p_*rec*.*dropout*_	0^*^	0.885 ± 0.003	0.727 ± 0.005
		0.1	0.876 ± 0.005	0.729 ± 0.011
		0.3	0.866 ± 0.003	0.733 ± 0.012
		0.5	0.857 ± 0.002	**0.740** ± 0.011
		0.7	0.852 ± 0.007	0.738 ± 0.017
		0.9	0.795 ± 0.057	0.733 ± 0.018
10	Conv. activation	leaky-relu	0.812 ± 0.129	0.660 ± 0.106
		relu^*^	0.885 ± 0.003	**0.727** ± 0.005
11	Rec. type	GRU	0.890 ± 0.007	0.726 ± 0.009
		LSTM^*^	0.885 ± 0.003	**0.727** ± 0.005
12	N_*CONVlayers*_	0	0.382 ± 0.067	0.344 ± 0.089
		1	0.752 ± 0.122	0.630 ± 0.078
		2	0.860 ± 0.014	0.722 ± 0.011
		3^*^	0.885 ± 0.003	**0.727** ± 0.005
13	N_*LSTMlayers*_	1	0.879 ± 0.005	0.718 ± 0.008
		2_*_	0.885 ± 0.003	0.727 ± 0.005
		4	0.855 ± 0.007	**0.729** ± 0.003
		8	0.430 ± 0.390	0.406 ± 0.366
14	N_*units*_	4	0.782 ± 0.008	0.723 ± 0.007
		8	0.803 ± 0.004	0.729 ± 0.010
		16	0.823 ± 0.008	**0.736** ± 0.007
		32	0.843 ± 0.005	0.731 ± 0.017
		64^*^	0.885 ± 0.003	0.727 ± 0.005
		128	0.906 ± 0.022	0.712 ± 0.007
		256	0.969 ± 0.001	0.708 ± 0.007
15	σ_*age*_	0	0.815 ± 0.125	0.642 ± 0.088
		1	0.882 ± 0.008	0.713 ± 0.015
		2	0.886 ± 0.004	0.720 ± 0.005
		3	0.879 ± 0.001	0.718 ± 0.004
		clamped^*^	0.885 ± 0.003	**0.727** ± 0.005
16	p_*gender*_	0	0.883 ± 0.005	**0.728** ± 0.011
		0.2	0.882 ± 0.008	0.726 ± 0.003
		0.4	0.885 ± 0.003	0.725 ± 0.008
		0.6	0.883 ± 0.004	0.717 ± 0.013
		0.8	0.876 ± 0.016	0.719 ± 0.002
		clamped^*^	0.885 ± 0.003	0.727 ± 0.005
17	Database	in-house^*^	0.885 ± 0.003	0.727 ± 0.005
		SIESTA	0.797 ± 0.001	**0.760** ± 0.022
18	Method	our^*^	0.885 ± 0.003	**0.727** ± 0.005
		log. reg.	0.422 ± 0.013	0.408 ± 0.041
		CNN [6]	0.820 ± 0.002	0.708 ± 0.005

*Kappa is shown as Mean ± STD*.

## References

[B1] Abadi M. Agarwal A. Barham P. Brevdo E. Chen Z. Citro C. (2016). Tensorflow: Large-scale machine learning on heterogeneous distributed systems. arXiv arXiv:1603.04467. Available online at: https://arxiv.org/abs/1603.04467

[B2] Biswal S. Kulas J. Sun H. Goparaju B. Westover M. B. Matt T. B. (2017). SLEEPNET: automated sleep staging system via deep learning. arXiv arXiv:1707.08262. Available online at: https://arxiv.org/abs/1707.08262

[B3] Boostani R. Karimzadeh F. Nami M. (2017). A comparative review on sleep stage classification methods in patients and healthy individuals. Comput. Methods Programs Biomed. 140, 77–91. 10.1016/j.cmpb.2016.12.004 28254093

[B4] Carrier J. Viens I. Poirier G. Robillard R. Lafortune M. Vandewalle G.. (2011). Sleep slow wave changes during the middle years of life. Eur. J. Neurosci. 33, 758–766. 10.1111/j.1460-9568.2010.07543.x 21226772

[B5] Carskadon M. A. Dement W. C. (2011). Chapter 2 - normal human sleep: an overview, in Principles and Practice of Sleep Medicine, 5th edn, eds Kryger M. H. Roth T. Dement W. C (St. Louis: Elsevier Saunders), 16–26.

[B6] Cho K. Van Merriënboer B. Bahdanau D. Bengio Y. (2014). On the properties of neural machine translation: Encoder-decoder approaches. arXiv arXiv:1409.1259. Available online at: https://arxiv.org/abs/1409.1259

[B7] Chollet F. (2017). Available online at: http://keras.io.

[B8] Cirelli C. Tononi G. (2008). Is sleep essential? PLoS Biol. 6:e216. 10.1371/journal.pbio.0060216 18752355 PMC2525690

[B9] Cohen J. (1960). A coefficient of agreement for nominal scales. Educ. Psychol. Meas. 20, 37–46. 10.1177/001316446002000104

[B10] Danker-Hopfe H. Anderer P. Zeitlhofer J. Boeck M. Dorn H. Gruber G.. (2009). Interrater reliability for sleep scoring according to the Rechtschaffen & Kales and the new AASM standard. J. Sleep Res. 18, 74–84. 10.1111/j.1365-2869.2008.00700.x 19250176

[B11] Estrada E. Nazeran H. Nava P. Behbehani K. Burk J. Lucas E. (2004). EEG feature extraction for classification of sleep stages, in Engineering in Medicine and Biology Society, 2004. IEMBS'04. 26th Annual International Conference of the IEEE, Vol. 1, (San Francisco, CA: IEEE), 196–199. 10.1109/IEMBS.2004.1403125 17271641

[B12] Fell J. Röschke J. Mann K. Schäffner C. (1996). Discrimination of sleep stages: a comparison between spectral and nonlinear EEG measures. Electroencephalogr. Clin. Neurophysiol. 98, 401–410. 10.1016/0013-4694(96)95636-9 8647043

[B13] Gal Y. Ghahramani Z. (2016). A theoretically grounded application of dropout in recurrent neural networks, in Advances in Neural Information Processing Systems, eds Pereira F. Burges C. J. C. Bottou L. Weinberger K. Q (Lake Tahoe, CA), 1019–1027.

[B14] Hobson J. A. (2005). Sleep is of the brain, by the brain and for the brain. Nature. 437, 1254–1256. 10.1038/nature04283 16251949

[B15] Hochreiter S. Schmidhuber J. (1997). Long short-term memory. Neural Comput. 9, 1735–1780. 10.1162/neco.1997.9.8.1735 9377276

[B16] Iber C. Ancoli-Israel S. Chesson A. Quan S. (2007). The AASM Manual for the Scoring of Sleep and Associated Events: Rules, Terminology, and Technical Specification. Westchester, IL.

[B17] Kingma D. Ba J. (2014). Adam: A method for stochastic optimization. arXiv arXiv:1412.6980. Available online at: https://arxiv.org/abs/1412.6980

[B18] Klosch G. Kemp B. Penzel T. Schlogl A. Rappelsberger P. Trenker E.. (2001). The SIESTA project polygraphic and clinical database. IEEE Eng. Med. Biol. Magaz. 20, 51–57. 10.1109/51.932725 11446210

[B19] Ko P. R. Kientz J. A Choe E. K Kay M. Landis C. A Watson N. F. (2015). Consumer sleep technologies: a review of the landscape. J. Clin. Sleep Med. 11, 1455–1461. 10.5664/jcsm.5288 26156958 PMC4661339

[B20] Krell M. M. Kim S. K. (2017). Rotational data augmentation for electroencephalographic data, in Engineering in Medicine and Biology Society (EMBC), 2017 39th Annual International Conference of the IEEE (Jeju). 10.1109/EMBC.2017.8036864 29059912

[B21] Krizhevsky A. Sutskever I. Hinton G. E. (2012). Imagenet classification with deep convolutional neural networks, in Advances in Neural Information Processing Systems, eds Pereira F. Burges C. J. C. Bottou L. Weinberger K. Q (Lake Tahoe, CA), 1097–1105.

[B22] Kuwahara H. Higashi H. Mizuki Y. Matsunari S. Tanaka M. Inanaga K. (1988). Automatic real-time analysis of human sleep stages by an interval histogram method. Electroencephalogr. Clin. Neurophysiol. 70, 220–229. 10.1016/0013-4694(88)90082-X 2458228

[B23] Landsness E. C. Goldstein M. R. Peterson M. J. Tononi G. Benca R. M. (2011). Antidepressant effects of selective slow wave sleep deprivation in major depression: a high-density EEG investigation. J. Psychiatr. Res. 45, 1019–1026. 10.1016/j.jpsychires.2011.02.003 21397252 PMC3119746

[B24] Maas A. L. Hannun A. Y. Ng A. Y. (2013). Rectifier nonlinearities improve neural network acoustic models. In Proc. ICML (Vol. 30, No. 1).

[B25] Marshall L. Helgadóttir H. Mölle M. Born J. (2006). Boosting slow oscillations during sleep potentiates memory. Nature 444, 610–613. 10.1038/nature05278 17086200

[B26] Marshall L. Mölle M. Hallschmid M. Born J. (2004). Transcranial direct current stimulation during sleep improves declarative memory. J. Neurosci. 24, 9985–9992. 10.1523/JNEUROSCI.2725-04.2004 15525784 PMC6730231

[B27] Nair V. Hinton G. E. (2010). Rectified linear units improve restricted Boltzmann machines, in Proceedings of the 27th International Conference on Machine Learning (ICML-10) (Haifa).

[B28] Ohayon M. M. Carskadon M. A. Guilleminault C. Vitiello M. (2004). Meta-analysis of quantitative sleep parameters from childhood to old age in healthy individuals: developing normative sleep values across the human lifespan. Sleep 27, 1255–1273. 10.1093/sleep/27.7.1255 15586779

[B29] Parks T. W. McClellan J. H. (1972). Chebyshev approximation for nonrecursive digital filters with linear phase. IEEE Trans. Circuit Theor. 19, 189–194. 10.1109/TCT.1972.1083419

[B30] Rolnick D. Veit A. Belongie S. Shavit N. (2017). Deep learning is robust to massive label noise. arXiv arXiv:1705.10694. Available online at: https://arxiv.org/abs/1705.10694

[B31] Srivastava N. Hinton G. E. Krizhevsky A. Sutskever I. Salakhutdinov R. (2014). Dropout: a simple way to prevent neural networks from overfitting. J. Mach. Learn. Res. 15, 1929–1958.

[B32] Supratak A. Dong H. Wu C. Guo Y. (2017). DeepSleepNet: a model for automatic sleep stage scoring based on raw single-channel EEG. IEEE Trans. Neural Syst. Rehabil. Eng. 99, 1–11. 10.1109/TNSRE.2017.2721116 28678710

[B33] Tsinalis O. Matthews P. M Guo Y. Zafeiriou S. (2016). Automatic sleep stage scoring with single-channel EEG using convolutional neural networks. arXiv arXiv:1610.01683v1. Available online at: https://arxiv.org/abs/1610.01683

[B34] Vogel G. W. Vogel F. McAbee R. S. Thurmond A. J. (1980). Improvement of depression by REM sleep deprivation: new findings and a theory. Arch. General Psychiatry 37, 247–253. 10.1001/archpsyc.1980.01780160017001 7362414

[B35] Zhu X. Bain M. (2017). B-CNN: branch convolutional neural network for hierarchical classification. arXiv arXiv:1709.09890. Available online at: https://arxiv.org/abs/1709.09890

